# Supplementary material for: A Global Screen for Assembly State Changes of the Mitotic Proteome by SEC-SWATH-MS
Source: Cell Syst. 2020 Feb 26;10(2):133–155.e6. doi: 10.1016/j.cels.2020.01.001 (PMC7042714; doi:10.1016/j.cels.2020.01.001)

Q5U5X0 | LYRM7_HUMAN | LYRM7 C5orf31 MZM1L
Monomer MW [kDa]: 11.955 Monomer expected elution fraction: 59

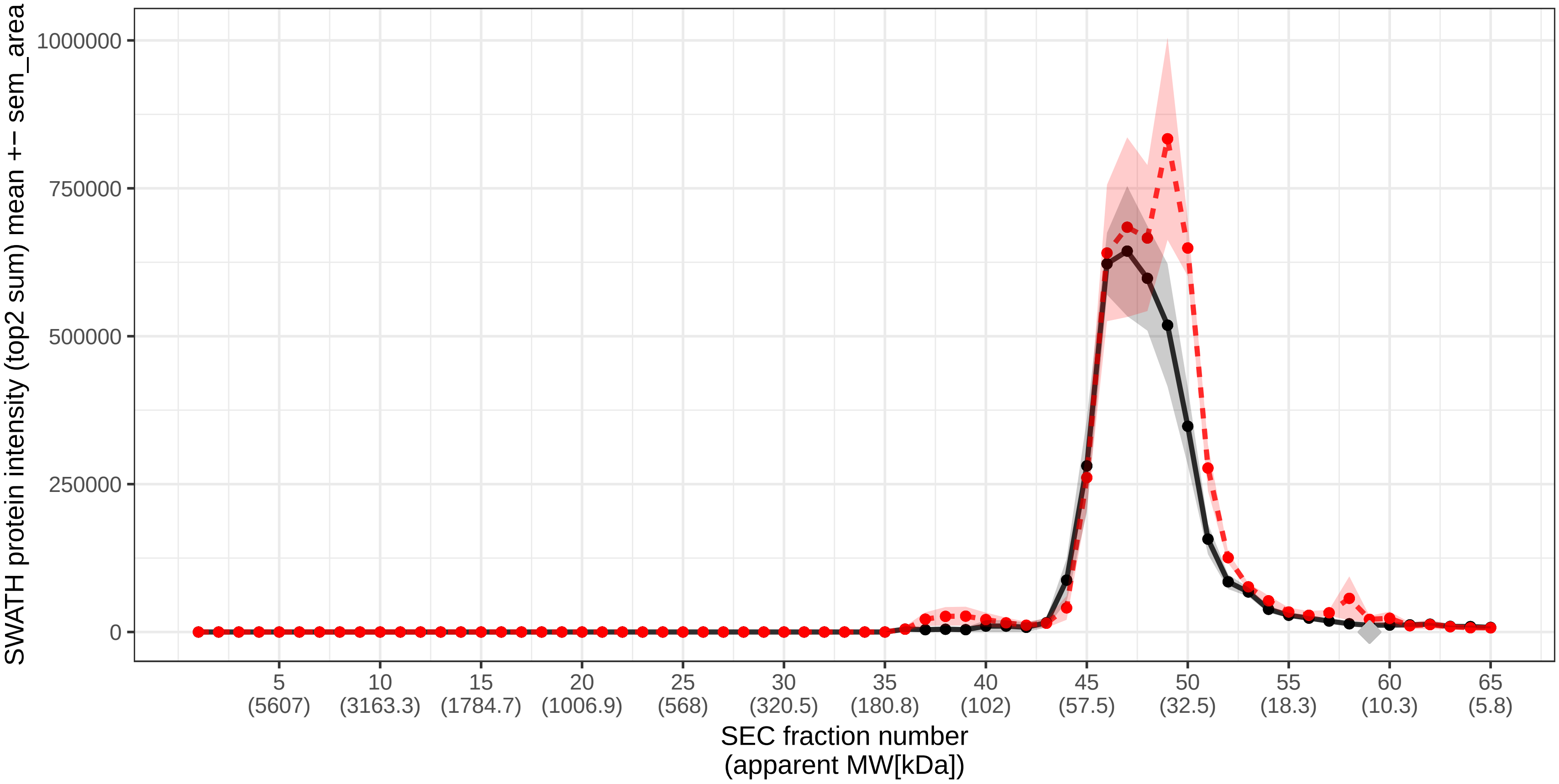

Supplement: Data S1. SEC-SWATH-MS Protein Chromatograms, Related to Figure 1 [file mmc6.zip › SECchrom_Q5U5X0_LYRM7_HUMAN_LYRM7_C5orf31_MZM1L.pdf]
